# Autotaxin May Have Lysophosphatidic Acid-Unrelated Effects on Three-Dimension (3D) Cultured Human Trabecular Meshwork (HTM) Cells

**DOI:** 10.3390/ijms222112039

**Published:** 2021-11-07

**Authors:** Megumi Watanabe, Masato Furuhashi, Yuri Tsugeno, Yosuke Ida, Fumihito Hikage, Hiroshi Ohguro

**Affiliations:** 1 Departments of Ophthalmology, School of Medicine, Sapporo Medical University, Sapporo 060-8556, Japan; watanabe@sapmed.ac.jp (M.W.); yuri.tsugeno@gmail.com (Y.T.); funky.sonic@gmail.com (Y.I.); fuhika@gmail.com (F.H.); 2 Departments of Cardiovascular, Renal and Metabolic Medicine, Sapporo Medical University, Sapporo 060-8556, Japan; furuhasi@sapmed.ac.jp

**Keywords:** 3D spheroid culture, human trabecular meshwork (HTM), autotaxin, lysophosphatidic acid

## Abstract

Purpose: The objective of the current study was to evaluate the effects of the autotaxin (ATX)–lysophosphatidic acid (LPA) signaling axis on the human trabecular meshwork (HTM) in two-dimensional (2D) and three-dimensional (3D) cultures of HTM cells. Methods: The effects were characterized by transendothelial electrical resistance (TEER) and FITC-dextran permeability (2D), measurements of size and stiffness (3D), and the expression of several genes, including extracellular matrix (ECM) molecules, their modulators, and endoplasmic reticulum (ER) stress-related factors. Results: A one-day exposure to 200 nM LPA induced significant down-sizing effects of the 3D HTM spheroids, and these effects were enhanced slightly on longer exposure. The TEER and FITC-dextran permeability data indicate that LPA induced an increase in the barrier function of the 2D HTM monolayers. A one-day exposure to a 2 mg/L solution of ATX also resulted in a significant decrease in the sizes of the 3D HTM spheroids, and an increase in stiffness was also observed. The gene expression of several ECMs, their regulators and ER-stress related factors by the 3D HTM spheroids were altered by both ATX and LPA, but in different manners. Conclusions: The findings presented herein suggest that ATX may have additional roles in the human TM, in addition to the ATX–LPA signaling axis.

## 1. Introduction

In the ectonucleotide pyrophosphatase/phosphodiesterase (ENPP) family, ENPP2 is also known as an autotaxin (ATX), a type of secreted glycoprotein [1,2,3]. It has been reported that ATX functions to hydrolyze lysophosphatidylcholine (LPC) to form lysophosphatidic acid (LPA) [4], and in turn, the resulting LPA interacts with six different G protein-coupled receptors (LPAR1–6) on the membrane, thus activating the coupled G proteins including Gq, Gi and G12/13 to facilitate their downstream signaling factors such as Rho, PLC, Ras, PI3K and others [5]. Therefore, this ATX/LPA/LPA receptor axis is known to be involved in a variety of physiological processes, including embryonic development, angiogenesis, and preadipocyte differentiation, as well as the pathogenesis of various types of cancer [6,7], neuropathic pain [8], and fat mass expansion [9]. It has also been suggested that this ATX/LPA/LPA receptor signaling may also contribute to the etiology of glaucoma, based upon elevated levels of LPA and ATX in the aqueous humor (AH) of patients with glaucoma [10,11,12,13,14,15], as well as mechanically stretched human TM (hTM) cells [15]. In addition to the above, it was also reported that the inhibition of ATX induced substantial hypotensive effects on intraocular pressure (IOP) in rabbits in vivo [15], and an ex vivo study using porcine or mice anterior segment organ cultures indicated a perfusion-induced aqueous outflow of LPA or ATX [15,16]. As a possible mechanism responsible for causing these ATX and LPA induced effects, the results of a conventional two-dimensional (2D) cell culture study using human trabecular meshwork (HTM) cells suggested that the ATX/LPA/LPA receptor signaling axis related to actin stress fibers, focal adhesions, and myosin light chain phosphorylation may be involved [10,11,17]. However, since such 2D cell cultures using HTM cells do not accurately reproduce the structures of multiple sheet layers of human TM [18], a system that replicates the 3D structure of the human TM, such as a relevant three-dimensional (3D) cell culture model, would be highly desirable. Our group recently reported on the development of a unique 3D cell drop culture system, and in our previous studies using this methodology [19,20,21,22], we were able to successfully produce 3D HTM spheroids that replicate the human TM multiple layer [23,24,25,26]. Furthermore, we also found that TGFβ2 induced the formation of significantly smaller and stiffer 3D HTM spheroids, and that such TGFβ2-induced effects were substantially reduced in the presence of Rho-associated coiled-coil containing protein kinase (ROCK) inhibitors [23,24]. These collective findings suggest that our 3D HTM spheroid system has the potential to serve as a physiologically relevant model that replicates human TM. This suggests that this model could be used in studies of the effects of the ATX/LPA/LPA receptor signaling axis on our 3D HTM spheroid.

In the current study, to pursue this issue in more detail, we examined the effects of ATX and LPA on the size, morphology and physical properties of the 3D spheroids and the expression of several genes including ECM molecules, their modulators including matrix metalloproteinase (MMP), tissue inhibitors metalloproteinase (TIMP), lysyl oxidase (LOX), and hypoxia inducible factor (HIF), and several endoplasmic reticulum (ER) stress-related factors.

## 2. Results

**Figure 1:**
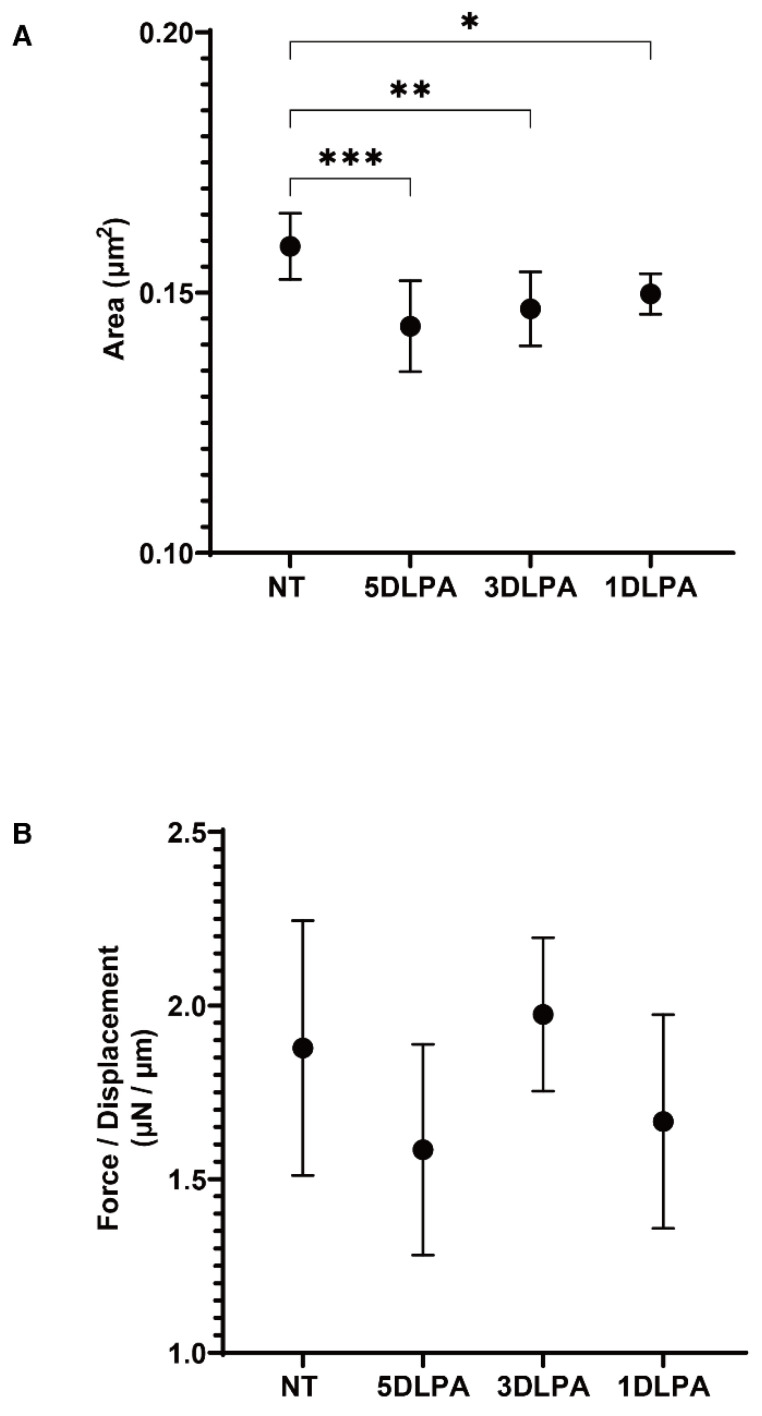
Effects of the exposure of different concentrations of LPA on the physical properties, size and stiffness of 3D HTM spheroids. During the 6-day culture of the 3D HTM spheroids, 200 nM LPA was exposed during Day 1 through 6 (5D LPA), Day 3 through 6 (3D LPA) or Day 5 through 6 (1D LPA). The mean sizes (**A**) and stiffness (**B**) of the HTM 3D spheroids under three different exposure conditions of fixed concentration of LPA (200 nM) were compared with non-treated controls with LPA (NT). Data are presented as the arithmetic mean ± standard error of the mean (SEM). * *p* < 0.05, ** *p* < 0.01, *** *p* < 0.005 (ANOVA followed by Tukey’s multiple comparison test).

To study the effects of LPA on human TM, the physical properties of the 3D HTM spheroids, which were previously established as a reliable in vivo model replicating human TM [23,24,25], were evaluated under several exposure periods (5 days; Day 1 through 6, 3 days; Day 3 through 6 or 1 day; Day 5 through 6) of 200 nM LPA during the 6-day culture. As shown in Figure 1, the mean sizes were significantly down-sized by exposure to LPA after a one-day exposure, but these down-sizing effects were slightly enhanced with longer exposures. However, in contrast, no significant effects by LPA were detected with respect to the stiffness of the 3D HTM spheroids.

**Figure 2:**
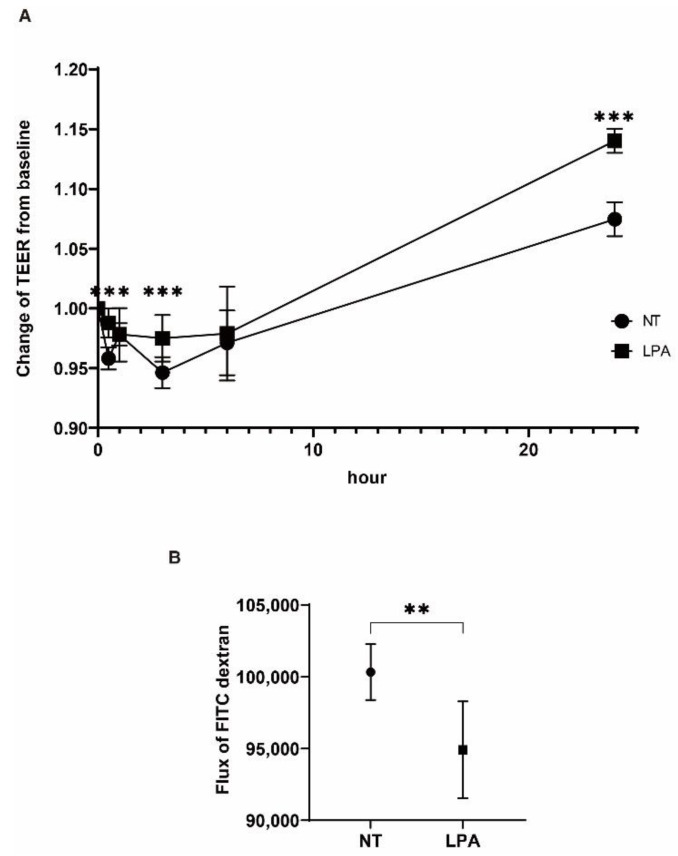
Effects of LPA on transendothelial electrical resistance (TEER) and FITC-dextran permeability of 2D cultures of HTM cell monolayers. Among the conditions in which the 2D cultured HTM monolayers were either untreated (NT) or treated with 200nM LPA, TEER (Ωcm^2^) values were measured at different time points (0, 0.5, 1, 3, 6 and 24 h) and plotted (**A**). Alternatively, 2D cultured HTM monolayers were either untreated (NT) or treated with 200 nM LPA for 24 h, and was FITC-dextran permeability was measured and the data plotted in (**B**). All experiments were performed in triplicate using fresh preparations (*n* = 4). Data are presented as the arithmetic mean ± standard error of the mean (SEM). ** *p* < 0.01, *** *p* < 0.005 (ANOVA followed by Tukey’s multiple comparison test).

To further examine the LPA-induced effects toward human TM, the barrier function of 2D cultured HTM cell monolayers was evaluated by transendothelial electron resistance (TEER). As shown in Figure 2A, the TEER values were substantially elevated by 200 nM LPA in a time-dependent manner during the 24-hour period of exposure, as compared to those of the non-treated control (NT). These data suggest that the presence of LPA induced an increase in the barrier functions of the 2D HTM monolayers, and this conclusion was also confirmed by the finding that a one-day exposure of LPA induced a substantial decrease in FITC-dextran permeability (Figure 2B). Therefore, these data indicate that a short time exposure of LPA rather that longer exposures caused significant effects on the 3D structure of the 3D spheroids as well as the barrier function of the 2D cultured HTM cells. In fact, rapid effects by LPA on these analyses were also reported by Nakamura et al. [27]. Therefore, these results and the above information related to 3D spheroid sizes provide a scientific rationale for the design of subsequent experimental protocols for LPA or ATX periods of exposure on the stiffness measurements and mRNA expression analyses of the 3D HTM spheroids.

**Figure 3:**
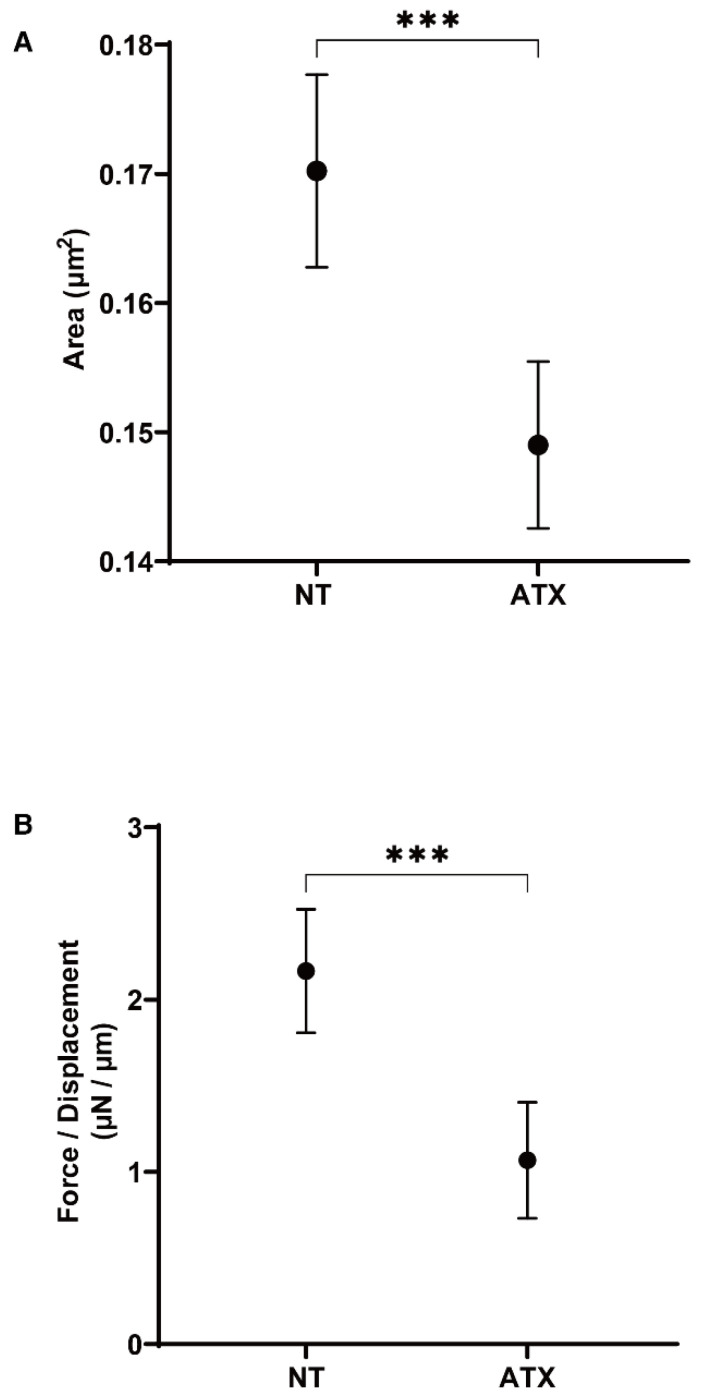
Effects of autotaxin (ATX) on the physical properties, size and stiffness of 3D HTM spheroid sizes. During the 6-day culture of the 3D HTM spheroids, they were exposed to a 2 mg/L solution of ATX during Day 5 through 6, and the mean sizes (µm^2^, panel (**A**)) and stiffness (µN/µm, panel (**B**)) of the HTM 3D spheroids were compared with non-treated control (NT). Data are presented as the arithmetic mean ± standard error of the mean (SEM). *** *p* < 0.005 (ANOVA followed by Tukey’s multiple comparison test).

**Figure 4:**
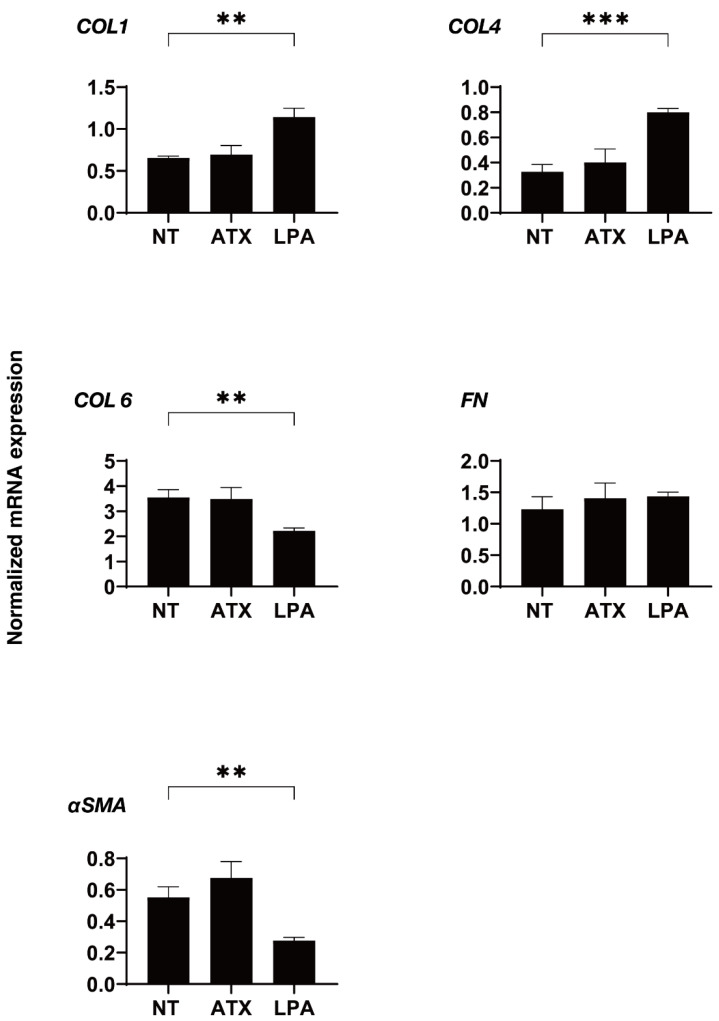
mRNA expression of ECM molecules in 3D cultured HTM cells. During the 6-day culture of the 3D HTM spheroids, 2 mg/L ATX or 200 nM LPA was exposed during Day 5 through 6, and the mRNA expression of ECMs (*COL1*, *COL4*, *COL6*, *FN* and *αSMA*) were measured. All experiments were performed in duplicate using fresh preparations. NT; untreated control. Data are presented as the arithmetic mean ± standard error of the mean (SEM). ** *p* < 0.01, *** *p* < 0.005 (ANOVA followed by Tukey’s multiple comparison test).

**Figure 5:**
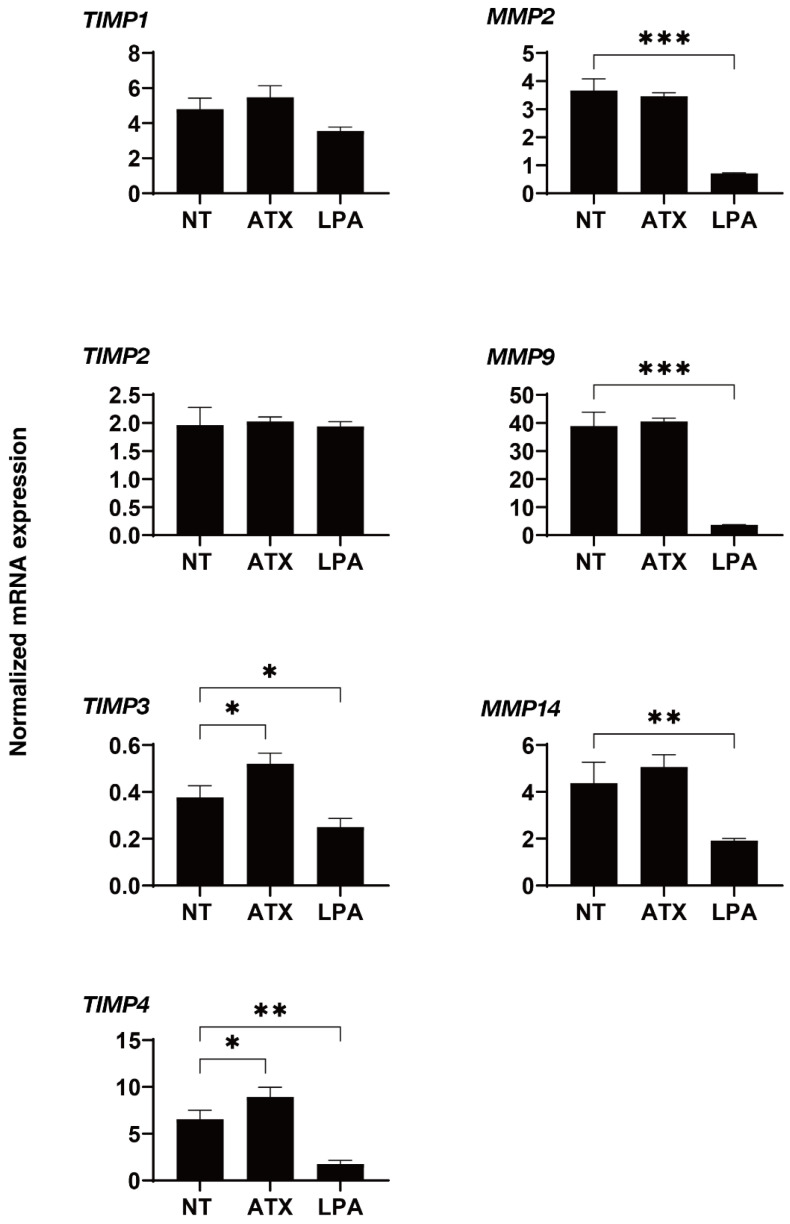
mRNA expression of TIMPs and MMPs in 3D cultured HTM cells. During the 6-day culture of the 3D HTM spheroids, 2 mg/L ATX or 200 nM LPA was exposed during Day 5 through 6, and the mRNA expression of *TIMP1-4*, and *MMP2*, *9* and *14* were measured. All experiments were performed in duplicate using fresh preparations. NT; untreated control. Data are presented as the arithmetic mean ± standard error of the mean (SEM). * *p* < 0.05, ** *p* < 0.01, *** *p* < 0.005 (ANOVA followed by Tukey’s multiple comparison test). TIMP; tissue inhibitor of metalloproteinase, MMP; matrix metalloproteinase.

**Figure 6:**
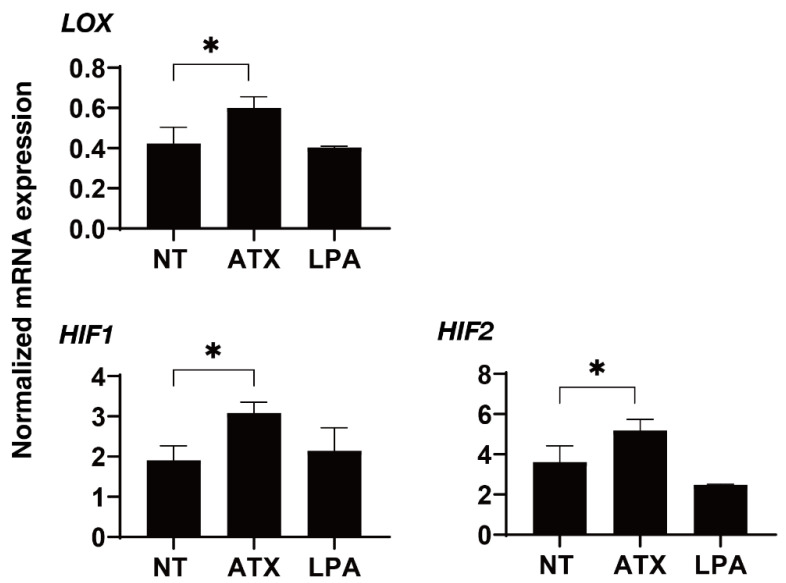
mRNA expression of LOX and HIFs in 3D cultured HTM cells. During the 6-day culture of the 3D HTM spheroids, 2 mg/L ATX or 200 nM LPA was exposed during Day 5 through 6, and the mRNA expression of *LOX*, and *HIF1* and *2* were measured. All experiments were performed in duplicate using fresh preparations. NT; untreated control. Data are presented as the arithmetic mean ± standard error of the mean (SEM). * *p* < 0.05, (ANOVA followed by Tukey’s multiple comparison test). LOX; lysyl oxidase, HIF; hypoxia inducible factor.

**Figure 7:**
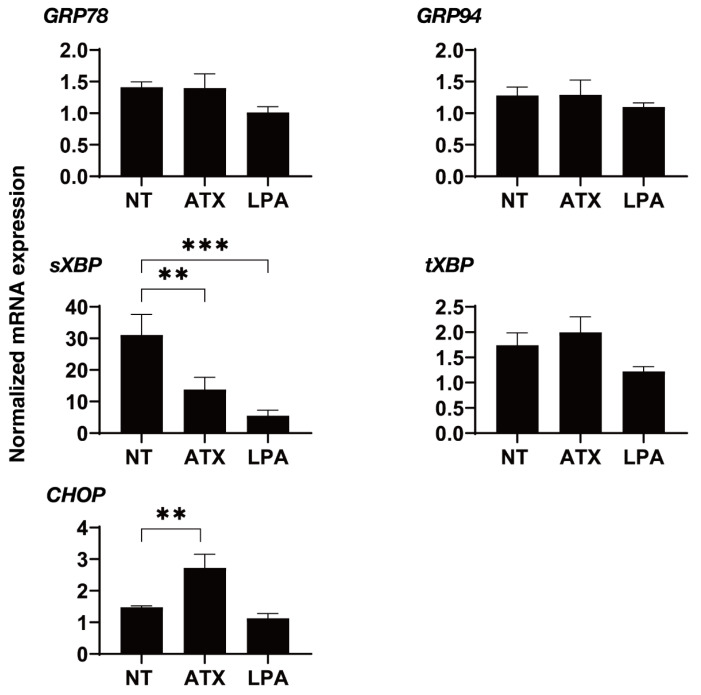
mRNA expression of ER-stress related genes in 3D cultured HTM cells. During the 6-day culture of the 3D HTM spheroids, they were exposed to 2 mg/L ATX or 200 nM LPA during Day 5 through 6, and the mRNA expression of ER-stress related genes including major ER stress related genes of inositol-requiring enzyme 1 (IRE1), glucose regulator protein (GRP)78, GRP94, the X-box binding protein-1 (XBP1), spliced XBP1 (sXBP1) and CCAAT/enhancer-binding protein homologous protein (CHOP) were measured. All experiments were performed in duplicate using fresh preparations. Data are presented as the arithmetic mean ± standard error of the mean (SEM). ** *p* < 0.01, *** *p* < 0.005 (ANOVA followed by Tukey’s multiple comparison test).

We next examined the effects of ATX, an up-stream regulatory factor of LPA, on the physical properties of the 3D HTM spheroids. Interestingly, as shown in Figure 3, a one-day exposure of ATX induced much stronger potent effects, as evidenced by the findings that the physical properties of the 3D HTM spheroids were significantly down-sized and less stiff as compared to spheroids that were exposed to LPA as above. To study this issue further, the effects induced by both ATX and LPA toward the expressions of several genes, including several ECM molecules (Figure 4), their regulators, TIMP, MMPs (Figure 5), LOX and HIFs (Figure 6), and several endoplasmic reticulum (ER)-stress related factors (Figure 7). Among these genes, LPA induced a significant up-regulation of *COL1* and *4* and the down-regulation of *COL6*, *αSMA*, *TIMP3* and *4*, *MMP2*, *9* and *14*, and *sXBP*, while ATX induced significant up-regulations of *TIMP3* and *4*, *LOX*, *HIF1* and *2*, and *CHOP*, and the down-regulation of *sXBP*. Therefore, these different effects between ATX and LPA regarding the expressions of several genes suggest that ATX may also play other roles, in addition to having an effect on the ATX–LPA signaling axis.

## 3. Discussion

Previous studies have demonstrated that several G-protein coupled receptors linked with both LPA and sphingosin-1-phosphate (S1P) are expressed in both TM as well in Schlemm’s canal (SC) cells [16,28]. In addition, the perfusion of LPA and S1P within enucleated eyes significantly influenced AH outflow, with concomitant changes in TM cell contractions, Rho GTPase activation and the expression of extracellular matrix proteins suggesting that these bioactive lipids have pivotal roles in the regulation of IOP levels by modulating the ease of AH outflow [16,28,29,30]. In fact, the following studies reported significantly increased concentrations of ATX [31] as well as their metabolite, LPA [32,33] within AH obtained from patients with glaucoma as compared with those with cataracts, but not glaucoma [15]. Furthermore, the AH ATX and LPA levels were also substantially different among several types of glaucoma, such as primary open-angle glaucoma (POAG), secondary glaucoma (SOAG) and exfoliative glaucoma (XFG) [14]. However, the magnitude of the AH levels varied substantially among the reported studies [14,15]. That is, Ho et al. reported that the ATX protein and LPA levels in AH from patients with primary open angle glaucoma (POAG) (ATX; 216.55 ± 15.37 ng/mL, LPA; 16:0; 11.3 ± 2.6 nM, 18:0; 3.4 ± 0.9 nM, 18:1; 10.2 ± 2.0 nM) were significantly increased as compared to patients with cataracts (ATX; 132.89 ± 6.67 ng/mL *p* < 0.001, LPA; 16:0; 1.3 ± 0.6 nM *p* < 0.001, 18:0; 3.5 ± 0.3 nM *p* = 0.825, 18:1; 3.5 ± 0.4 nM *p* = 0.003) [15]. In another study, Honjo and associates reported that the values for the total ATX and LPA were approximately 0.5–2 µg/mL and 40–200 nM, respectively, although these values varied with the type of glaucoma [13,14]. Therefore, these data suggest that the ATX/LPA signaling axis may be heavily involved in the pathogenesis of glaucoma, although the reason for such diversity of these AH concentrations between groups has not yet been elucidated.

To study this issue, Honjo and associates extended their research to include the use of 2D HTM cultures [12,27,34]. Among these studies, they reported that ATX and LPA consistently induced a significant up-regulation of fibrogenic factors including COL1, FN and αSMA. Furthermore, the genes responsible for such ATX- and LPA-induced effects showed a time-dependent difference in their upregulation [27]. That is, (1) COL1 was significantly up-regulated only after 6 h, but those up-regulations were not observed after 24 h; (2) αSMA was significantly up-regulated by LPA during 6 to 24 h, and ATX after 24 h; and (3) a significant up-regulation of FN by ATX was observed only after 1 h, but no significant changes were observed by LPA during a 24 h period of incubation [27]. However, if ATX is actually an up-stream regulator of LPA, as is currently well known [35], effects by exogenous administration should be evident earlier for LPA compared to ATX, i.e., the earlier up-regulation of FN by ATX cannot be explained. As a possible reason for this, the very high concentrations of ATX (40 µM) or LPA (10 µM) that were used for these 2D cultured HTM experiments as compared to their physiological concentrations detected from patients’ specimens (ATX; 0.5–0.8 nM, LPA; 10–200 nM) may be related. In the current study, to examine the effects of ATX or LPA on the human TM, we used 3D HTM spheroids, a recently established physiologically relevant in vivo model for human TM [23,24,25,26]. The findings indicate that LPA and ATX exerted substantial down-sizing effects on 3D HTM spheroids, but the nature of these effects, including stiffness and the gene expressions of several related factors including ECMs, ECM regulatory factors and ER-stress related genes, was distinctly different (Figure 4, Figure 5, Figure 6 and Figure 7). Among these, a significant up-regulation of HIF1 and 2 was observed for ATX but not for LPA (Figure 6). Interestingly, a previous study using an ATX knockout mouse demonstrated the absence of HIF1 expression [36]. Therefore, the collective findings reported herein strongly suggest that, based on findings regarding 3D HTM spheroids, that ATX has additional roles beyond its role in the ATX–LPA–LPA receptor signaling axis [35].

During the ATX–LPA–LPA receptor signaling axis, LPA is delivered to the surface of the target cell, where it binds to LPAR1-6. The LPA then activates different downstream signal cascades, including Rho, phospholipase C (PLC), mitogen-activated protein kinase (MAPK), phosphatidylinositide 3-kinase (PI3K), and adenylate cyclase (AC), through different G proteins [35]. LPA is mainly produced by the ATX-mediated hydrolysis of lysophosphatidylcholine (LPC) and, in turn, is degraded to monoacylglycerol (MAG) through the actions of a lipid phosphate phosphohydrolase (LPP1-3). On the other hand, the ATX protein is composed of a central phosphodiesterase (PDE) domain, a C-terminal nuclease (NUC)-like domain and two N-terminal somatomedin B (SMB)-like domains. Among these, the SMB-like domain functions to bind with cell surface integrin for the stimulation of LPA to its receptors [35]. We therefore speculate that this binding of ATX to the cell surface integrin may cause additional unknown pathophysiological effects other than those on the main ATX–LPA–LPA receptor signaling axis. In fact, we detected the presence of ATX and LPA receptors in 3D HTM spheroids (Supplemental Figure S1). In addition, if this ATX–LPA–LPA receptor signaling axis is actually involved in the pivotal roles related to AH outflow including TM, those effects should be continuous. However, a previous study by Honjo and associates reported that the administration of ATX or LPA to 2D HTM or Schlemm’s canal endothelial (SCH) cells caused significant biological effects during an initial short period, and these effects then decayed substantially, although TGFβ, an established modulator that induces fibrogenic changes toward the AH outflow pathway [37,38,39,40], was continuously affected [27]. In fact, similar transient effects were also observed in our current study in the case of LPA administration. As another possibility, LPA receptor distribution—that is, the predominant expression of LPA6 receptors, less expression of LPA1-3 receptors and no expression of LPA4 and 5 receptors within HTM cells—may be involved (Supplemental Figure S1).

In conclusion, based upon our current study, we hypothesize that the unrelated effects of ATX with LPA can be attributed to the binding of ATX to cell surface integrins via the SMB domains. However, as of writing this, to prove this hypothesis, additional investigations will be needed, including (1) the effects of exogenous treatment on the catalytically inactive ATX enzyme (mutation at the catalytic site T210A) [41], (2) the effects of phosphatase on the breakdown of LPA, and (3) the roles of several species of LPAs (18:1, 18:0, 16:0, etc.) within HTM cells, and others. These represent future projects.

## 4. Materials and Methods

### 4.1. 3D Spheroid Cultures of Human Trabecular Meshwork (HTM) Cells

Immortalized human trabecular meshwork (HTM) cells that were obtained from Applied Biological Materials Inc., Richmond, BC, Canada were used in the present study. Three-dimensional cultures of the HTM cells were basically carried out as described previously [23,24,25,26]. Briefly, 2D cultured HTM cells were further processed into 3D spheroid cultures on a hanging droplet spheroid (3D) culture plate (# HDP1385, Sigma-Aldrich, St. Louis, MO, USA) over a period of 6 days. For evaluating drug induced efficacies, 200 nM LPA was added at Days 1, 3 or 5 or, alternatively, 2 mg/L ATX was added at Day 5, and the resulting 3D cultures were maintained by changing half volumes of the medium (14 μL) each day in each well. These concentrations of LPA and ATX were followed by maximum AH concentrations that were detected from several glaucoma patients in a previous study [14]. As shown in Supplemental Figure S1, ATX receptors and LPA receptors were expressed in the 3D HTM spheroid used in the current study. However, among the 6 LPA receptors, predominant expression of the LPA6 receptor, less expression of LPA1-3 receptors and no expression of LPA4 and 5 were detected.

### 4.2. Measurement of the Physical Properties, Size and Stiffness of the 3D HTM Spheroids

As described previously, the configurations of the 3D spheroids were observed by phase contrast microscopy (PC, Nikon ECLIPSE TS2; Tokyo, Japan), and the mean size of each 3D spheroid, defined as their largest cross-sectional area (CSA), were calculated using the Image-J software version 1.51n (National Institutes of Health, Bethesda, MD, USA) [23,24,25,26].

The physical stiffness analysis of the 3D HTM spheroids was measured using a micro-squeezer (MicroSquisher, CellScale, Waterloo, ON, Canada) as previously reported [23,24,25,26]. Briefly, the force (μN/μm) required to compress a single spheroid on a 3 mm square plate to a deformation of 50% during a 20 s interval was measured.

### 4.3. Quantitative PCR

Total RNA extraction, reverse transcription and subsequent real-time PCR with the Universal Taqman Master mix using a StepOnePlus instrument (Applied Biosystems/Thermo Fisher Scientific) were performed as described previously [23,24,25,26]. The respective cDNA values are shown as fold-change relative to the normalized housekeeping gene 36B4 (*Rplp0*), as a control. Sequences of primers and Taqman probes used are listed in supplemental Table S1.

### 4.4. Statistical Analysis

Through statistical analyses using Graph Pad Prism 8 (GraphPad Software, San Diego, CA, USA), statistical significance with a confidence level greater than 95% by means of a two-tailed Student’s t-test or two-way analysis of variance (ANOVA) followed by Tukey’s multiple comparison test was performed as described previously [23,24,25,26].

## Data Availability

The data that support the findings of this study are available from the corresponding author upon reasonable request.

## Supplementary Materials

The following are available online at https://www.mdpi.com/article/10.3390/ijms222112039/s1.
