# Supplementary material for: Autotaxin May Have Lysophosphatidic Acid-Unrelated Effects on Three-Dimension (3D) Cultured Human Trabecular Meshwork (HTM) Cells
Source: Int J Mol Sci. 2021 Nov 7;22(21):12039. doi: 10.3390/ijms222112039 (PMC8584821; doi:10.3390/ijms222112039)
Supplement: Supplementary file 1 [file ijms-22-12039-s001.zip › ijms-1443014-supplementary.pdf]

Supplemental Table S1 Sequences of primers used in the qPCR

		Sequence	Exon Location	RefSeq Number
human RPLP0	Probe<br>Primer2<br>Primer1	5'-/56-FAM/CCCTGTCTT/ZEN/CCCTGGGCATCAC/3IABkFQ/-3'<br>5'-TCGTCTTTAAACCCTGCGTG-3'<br>5'-TGTCTGCTCCCACAATGAAAC-3'	2-3	NM_001002
human COL1A1	Probe<br>Primer2<br>Primer1	5'-/56-FAM/TCGAGGGCC/ZEN/AAGACGAAGACATC/3IABkFQ/-3'<br>5'-GACATGTTCAGCTTTGTGGAC-3'<br>5'-TTCTGTACGCAGGTGATTGG-3'	1-2	NM_000088
human COL4A1	Probe<br>Primer2<br>Primer1	5′-/56-FAM/TCATACAGA/ZEN/CTTGGCAGCGGCT/3IABkFQ/-3′<br>5′-AGAGAGGAGCGAGATGTTCA-3′<br>5′-TGAGTCAGGCTTCATTATGTTCT-3′	51-52	NM_001845
human COL6A1	Primer2<br>Primer1	5'-CCTCGTGGACAAAGTCAAGT-3'<br>5'-GTGAGGCCTTGGATGATCTC-3'	2-3	NM_001848
human FN1	Primer2<br>Primer1	5'-CGTCCTAAAGACTCCATGATCTG-3'<br>5'-ACCAATCTTGTAGGACTGACC-3'	3-4	NM_212482
human αSMA	Probe<br>Primer2<br>Primer1	5'-/56-FAM/AGACCCTGT/ZEN/TCCAGCCATCCTTC/3IABkFQ/-3'<br>5'-AGAGTTACGAGTTGCCTGATG-3'<br>5'-CTGTTGTAGGTGGTTTCATGGA-3'	8-9	NM_001613
human TIMP1	Probe<br>Primer2<br>Primer1	5′-/56-FAM/TCAACCAGA/ZEN/CCACCTTATACCAGCG/3IABkFQ/-3′<br>5′-CCTTCTGCAATTCCGACCT-3′<br>5′-GCTTGGAACCCTTTATACATCTTG-3′	2-4	NM_003254
human TIMP2	Probe<br>Primer2<br>Primer1	5′-/56-FAM/TCTCATTGC/ZEN/AGGAAAGGCCGAGG/3IABkFQ/-3′<br>5′-GACGTTGGAGGAAAGAAGGA-3′<br>5′-TGTGGTTCAGGCTCTTCTTC-3′	3-4	NM_003255
human TIMP3	Probe<br>Primer2<br>Primer1	5′-/56-FAM/CCTCCTTTA/ZEN/CCAGCTTCTTCCCCAC/3IABkFQ/-3′<br>5′-CCTTCTGCAACTCCGACATC-3′<br>5′-CGGTACATCTTCATCTGCTTGA-3′	1-3	NM_000362
human TIMP4	Probe<br>Primer2<br>Primer1	5′-/56-FAM/ACTGAGGAC/ZEN/CTGACCAGTCAAGAGA/3IABkFQ/-3′<br>5′-GGTTTGAGAAAGTCAAGGATGTTC-3′<br>5′-GTTGCACAGATGGATGAAGAC-3′	3-4	NM_003256
human MMP2	Primer2<br>Primer1	5′-TCCACCACCTACAACTTTGAG-3′<br>5′-GTGCAGCTGTCATAGGATGT-3′	6-7	NM_004530
human MMP9	Primer2<br>Primer1	5′-ACATCGTCATCCAGTTTGGTG-3′<br>5′-CGTCGAAATGGGCGTCT-3′	3-4	NM_004994
human MMP14	Primer2<br>Primer1	5′-TTCGCCGACTAAGCAGAAG-3′<br>5′-CTTGAATTCCTAGACCGCTGT-3′	1-1	NM_004995
human LOX	Primer2<br>Primer1	5′-ACATTCGCTACACAGGACATC-3′<br>5′-TTCCCACTTCAGAACACCAG-3′	6-7	NM_002317
human HIF1A	Primer2<br>Primer1	5′-CTCTGATCATCTGACCAAAACTCA-3′<br>5′-CAACCCAGACATATCCACCTC-3′	8-9	NM_181054
human HIF2A	Primer2<br>Primer1	5′-AGCCTATGAATTCTACCATGCG-3′<br>5′-CTTTGCGAGCATCCGGTA-3′	7-8	NM_001430
human GRP78	Forward<br>Reverse	5'-CATCACGCCGTCCTATGTCG-3'<br>5'-CGTCAAAGACCGTGTTCTCG-3'		NM_005347
human GRP94	Forward<br>Reverse	5'-CTGGGACTGGGAACTTATGAATG-3'<br>5'-TCCATATTCGTCAAACAGACCAC-3'		NM_003299

human sXBP	Forward<br>Reverse	5'-GGTCTGCTGAGTCCGCAGCAGG-3'<br>5'-GGGCTTGGTATATATGTGG-3'		AB076384
human tXBP	Forward<br>Reverse	5'-AGTAGCAGCTCAGACTGCCA-3'<br>5'-CCTGGTTCTCAACTACAAGGC-3'		NM_005080
human CHOP	Forward<br>Reverse	5'-GGAGAACCAGGAAACGGAAAC-3'<br>5'-TCTCCTTCATGCGCTGCTTT-3'		NM_004083
human ATX	Primer2<br>Primer1	5′-GGAGAAACACGGACATCAGG-3′<br>5′-GCCACTCTGGACATCATATACTG-3′	23-24	NR_045555
human LPAR1	Primer2<br>Primer1	5′-ACAGTGATTCCAAGTCCCATC-3′<br>5′-GTAATTTCACAGCCCCAGTTC-3′	3-4	NM_057159
human LPAR2	Primer2<br>Primer1	5′-GTACTTTTCTACAGCCAGGACA-3′<br>5′-AGCCTGGTCAAGACTGTTG-3′	2-3	NM_004720
human LPAR3	Primer2<br>Primer1	5′-AGCAGCAGGAACCACCT-3′<br>5′-ACCCATGAAGCTAATGAAGACG-3′	2-3	NM_012152
human LPAR4	Primer2<br>Primer1	5′-GGTTCACCACTCTGACACTATG-3′<br>5′-TCTACAGGCATCAGCACATTC-3′	1-2	NM_005296
human LPAR5	Primer2<br>Primer1	5′-GTCATGGGAATGTGGGCTAT-3′<br>5′-AGAGCAACACGGAGCAC-3′	1-3	NM_020400
human LPAR6	Primer2<br>Primer1	5′-CCTCCAGCAAATTCCAGCA-3′<br>5′-GGTACAATCAAAGCTCACTGC-3′	4-5	NM_005767

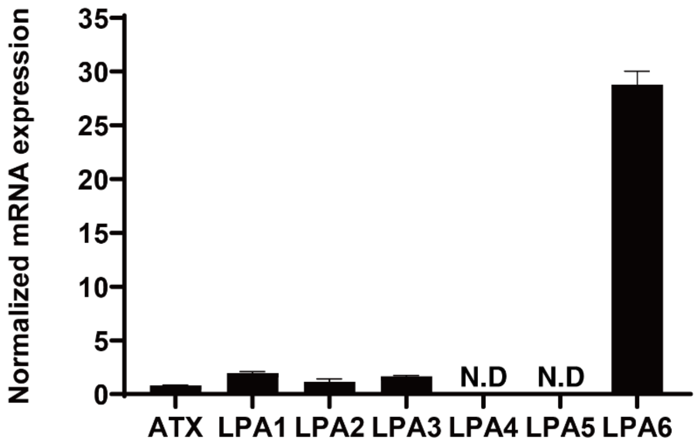

Figure S1. mRNA expression of ATX receptor and LPA 1-6 receptors in 3D HTM spheroid. 3D HTM spheroids at Day 6 were subjected to qPCR analysis to estimate the expression of the mRNA of ATX receptor and LPA 1-6 receptors. All experiments were performed in duplicate using fresh preparations. Data are presented as the arithmetic mean ± standard error of the mean (SEM).
